# Unveiling the awareness deficits related to tobacco and prevention measures in patients with oral cancer in India: a cross-sectional study

**DOI:** 10.3332/ecancer.2025.2004

**Published:** 2025-10-06

**Authors:** Parth Sharma, Mongjam Meghachandra Singh, Amod Laxmikant Borle, Anurita Srivastava, Ravi Meher

**Affiliations:** 1 Department of Community Medicine, Maulana Azad Medical College, Delhi 110002, India; 2 Department of Radiation Therapy, Maulana Azad Medical College, Delhi 110002, India; 3 Department of E.N.T, Maulana Azad Medical College, Delhi 110002, India

**Keywords:** tobacco, oral cancer, cancer prevention, public health, health policy, awareness

## Abstract

**Introduction:**

Oral cancer is a major public health issue in India. Despite various prevention strategies, the incidence of oral cancer continues to rise. This study aimed to assess awareness of tobacco’s harmful effects and tobacco control measures in patients with oral cancer.

**Methods:**

This cross-sectional study was conducted from August 2023 to June 2024. Totally, 116 adult patients with histopathologically confirmed oral cancer were recruited using convenience sampling. Data were collected using a pretested interview schedule covering sociodemographics, tobacco and alcohol use, awareness of oral cancer and tobacco control policies. Descriptive statistical methods were used to analyse the data, and chi-Square and Fisher’s Exact tests were used to identify factors associated with awareness of the carcinogenic nature of tobacco.

**Results:**

The mean age of participants was 47.9 ± 11.1 years, with 87.9% male and 97.4% from upper, lower/lower socioeconomic backgrounds. 54.3% of participants used smokeless tobacco, 10.3% were smoking and 27.6% were using both. Most consumed tobacco daily, with 52.6% quitting tobacco consumption after getting diagnosed with oral cancer. 66.4% were aware of the link between tobacco and oral cancer, primarily from tobacco packaging (48.1%) and anti-tobacco advertisements (36.3%). However, all were unaware of the early symptoms of oral cancer and self-examination methods. Awareness of free government screenings was very low (0.9%), and only 7.8% knew of laws regulating tobacco. Awareness that tobacco causes cancer was significantly higher among literate participants, those who noticed the warning sign, and felt fear from the warning signs on tobacco products (p < 0.05).

**Conclusion:**

This study reveals significant gaps in awareness regarding tobacco-related oral cancer risks and preventive measures among patients with oral cancer. Targeted awareness campaigns and improved access to screening could help reduce oral cancer in India.

## Introduction

Oral cancer and oropharyngeal cancers (hereafter referred to collectively as ‘oral cancer’) are preventable diseases. India reports the highest burden of oral cancer globally. However, despite having a law regulating tobacco production and sale, a national program for tobacco control and prevention and a national program for oral cancer prevention and screening in place, the burden of oral cancer continues to rise in India [1, 2].

The National Family Health Survey 5 Report indicated that less than 1% of the population was screened for oral cancer in India between 2019 and 2021 [3]. This low screening rate, coupled with inadequate awareness among both healthcare workers and patients regarding early signs and symptoms of oral cancer, has led to delays in the diagnosis of oral cancer. According to the National Cancer Registry Program Report 2020, nearly two-thirds of oral cancer patients in India were diagnosed at advanced stages of the disease [4].

While the awareness of the harmful effects of tobacco has been studied in the general population, literature on its awareness among patients with oral cancer is lacking. Understanding the awareness levels of this population will help in modifying the current approach to oral cancer prevention by addressing the gaps identified. This study aims to assess the awareness of tobacco-related harmful effects and government-led tobacco control measures in patients with oral cancer.

## Methods

### Study design and study population

This cross-sectional study was conducted at the Lok Nayak Hospital in Delhi from August 2023 to June 2024. Patients above 18 years of age with a histopathologically confirmed diagnosis of oral cancer were included in the study. Severely ill patients and patients with a history of cancer of other body parts or with recurrence of oral cancer were excluded from the study. This study was a part of a larger project assessing delays in the diagnosis and treatment of patients with oral cancer [5]. Ethical clearance was taken from the Institutional Ethical Committee (F.1/IEC/MAMC/MD/MS(96/02/2023/No.125) on 15.05.2023.

### Sampling method and data collection

Convenient sampling was done to collect data from oral cancer patients presenting to the outpatient department. A pretested interview schedule was used to gather the information from the participants. Written informed consent was taken from all individuals willing to participate in the study. The data was collected using EpiCollect5 software.

### Statistical analysis

Descriptive statistical methods were used to summarise categorical and continuous variables. Mean and standard deviation were used for variables with a Gaussian distribution, and median and interquartile range (IQR) for variables with a skewed distribution. Combined pack years for cigarettes and bidis were calculated using the methodology proposed by Datta *et al* [6]. Chi-Square and Fisher’s Exact test were used to identify factors associated with awareness that tobacco causes cancer. Significance levels were defined as a *p*-value less than 0.05. All analysis was done in SPSS Ver 25.0.

## Results

### Sociodemographic details

A total of 116 patients with oral cancer were included in this study. The mean age of the study population was 47.9 ± 11.1 years, with 92 (79.3%) participants under the age of 60 years. The majority were male (102, 87.9%) and 39 (33.6%) were illiterate. The median (IQR) per capita family income was 17.04 USD (0, 29.10), with 36 (31.0%) families having no monthly income. A majority were unemployed (104, 89.7%) and belonged to the upper lower or lower socioeconomic class (113, 97.4%). Internet access was present only in 44 (37.9%) study participants.

### Personal history of study participants

Among the study participants, 63 (54.3%) had a history of smokeless tobacco use, 12 (10.3%) had a history of smoking tobacco, 32 (27.6%) had a history of consuming both and 9 (7.8%) gave a history of never consuming tobacco. Out of those who smoked tobacco (*n* = 44), 41 (93.2%) smoked bidis and only 3 (6.8%) smoked cigarettes. The frequency of smoking (*n* = 44) was daily for 41 (93.2%) and 3 (6.8%) smoked more than once a week but not daily. The median (IQR) pack years smoked were 5.5 (1.9–8.8) years. The frequency of consumption of smokeless tobacco for those consuming (*n* = 95) was daily for 87 (91.6%) participants, while 5 (5.3%) consumed it more than once a week but not daily and 3 (3.1%) consumed it less than once a week. At the time of the interview, 2 (1.7%) of the participants were consuming tobacco, 14 (12.1%) had quit before experiencing symptoms, while 30 (25.9%) quit after symptoms appeared. The majority, 61 (52.6%), stopped using tobacco after being diagnosed with oral cancer.

For alcohol, 60 (51.7%) reported consumption, with 56 (48.3%) participants with no history of alcohol consumption. Of those who consumed alcohol (*n* = 60), 30 (50.0%) consumed it daily, while 14 (23.3%) consumed it more than once a week but not daily and 16 (26.7%) consumed alcohol less than once a week.

### Disease-related awareness in the study population

Awareness of the relationship between tobacco use and oral cancer was present in 77 (66.4%) participants, while 39 (33.6%) were unaware. The primary sources of information (*n* = 77) included tobacco packaging (37, 48.1%), anti-tobacco advertisements (28, 36.3%) and friends (11, 14.3%). The healthcare provider was reported to be the source of this information by only 1 (1.3%) participant. Notably, all participants (100%) were unaware of the existence of a national program for oral cancer. All participants (100%) were unaware of the early symptoms of oral cancer.

### Awareness of tobacco and oral cancer control measures in India

**Figure 1.**
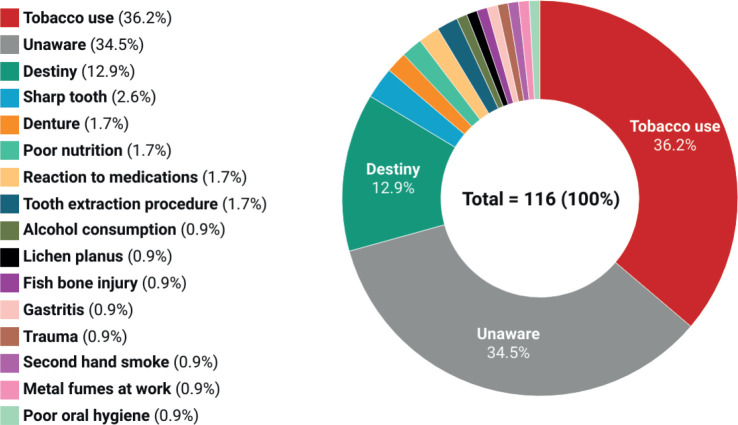
Patient-attributed causes of their oral cancer.

Only 1 (0.9%) participant was aware of free oral cancer screenings in government healthcare centers. All (100%) participants were unaware of self-examination for oral cancer and had never been referred for oral cancer screening to a healthcare facility. Only 9 (7.8%) were aware of the existence of laws governing tobacco production and sale in India. Most participants (97, 83.6%) had noticed warning signs on tobacco products, but only 44 (37.9%) expressed fear in response to these warnings. Only 42 (36.2%) participants attributed their cause of cancer to tobacco use (Figure 1).

Notably, a higher percentage of people less than 60 years of age and males were aware that tobacco caused cancer. However, these differences were not statistically significant (*p* <0.05). Similarly, compared to those who smoked tobacco, a higher percentage of people who consumed smokeless tobacco were aware that tobacco caused cancer (*p* > 0.05). Socioeconomic class and employment status were also not significantly associated with the presence of awareness that tobacco caused cancer (*p* > 0.05). Literate people were more likely to be aware of the cancer-causing nature of tobacco compared to illiterate participants (*p* = 0.014). People who reported noticing the warning sign on tobacco products (*p* = 0.001) and those who reported feeling fear after noticing the warning sign (*p* = 0.046) were also more likely to be aware that tobacco caused cancer. A higher percentage of people who attributed tobacco use as their cause of cancer were also aware that tobacco caused cancer. However, this association was not statistically significant (*p* = 0.160).

## Discussion

In our study, we found consumption of smokeless tobacco to be more prevalent than the smoked form of tobacco. Among those who smoked tobacco, the consumption of bidi was much more prevalent than the consumption of cigarettes. Globally, smokeless tobacco consumption contributes to one in three cases of oral cancer [7]. Similar to other studies from India, our study showed that a greater proportion of patients were chewing tobacco and smoking bidis [8]. These two forms of tobacco are known to be associated with a higher risk of oral cancer as compared to cigarettes [8]. As these products are also considerably cheaper compared to cigarettes, their high proportion of consumption can be explained in our study population as the majority belonged to upper lower or lower socioeconomic status.

Most participants (66.4%) were aware of the link between tobacco use and oral cancer. While others knew tobacco caused harm to health, they were not aware that it led to oral cancer. This could be due to the warning signs, which currently mention ‘Tobacco Causes Painful Death’ but do not mention the link between oral cancer and tobacco use [9]. Even though the graphic warning currently being used in India shows a malignant oral cancer lesion, various studies have reported that these images are difficult to interpret due to their poor quality [10].

We also found that all patients were unaware of early symptoms and the existence of a national program that focuses on the prevention of oral cancer. Only a small percentage (0.9%) knew about free oral cancer screenings, and only 7.8% were aware of tobacco regulations in India. Similar to this study, the awareness of oral cancer has been found to be inadequate in studies published from other parts of India [11, 12]. The lack of awareness is the possible reason for poor coverage of oral cancer screening in India (<1%) despite there being a national program focusing on oral cancer prevention [3]. While 83.6% noticed warnings on tobacco products, only 37.9% reported feeling fear in response. These highlight the questionable role of the pictorial warnings on tobacco products in increasing awareness related to the harmful effects of tobacco.

As tobacco packaging was reported to be the most common source of information related to the harmful effects of tobacco, there is a need to review the messages put on the packaging for more effective communication. Our study highlights that people who noticed the warning sign on the tobacco product were more likely to be aware that tobacco caused oral cancer. Surprisingly, only one patient reported a healthcare provider to be the source of information for the harmful effects of tobacco. Similarly, none of the patients were aware of self-oral examination and had never been advised to get screened for oral cancer. This highlights the need for healthcare providers to engage in health promotion activities and conduct opportunistic screening for individuals with a history of tobacco consumption seeking care at a healthcare setup.

### Policy recommendations

Our study highlights critical gaps in awareness regarding the link between tobacco use and oral cancer, the availability of government-led screening programs and early symptoms of the disease. To address these knowledge gaps, a multi-pronged policy approach is recommended. First, public health campaigns should be intensified to communicate the specific risk of oral cancer from tobacco use. Pictorial warnings on tobacco products must be improved in terms of clarity and should explicitly depict oral cancer lesions with accompanying text in local languages and not only in English and Hindi. Community-based interventions such as awareness drives, street plays and group education sessions led by ASHAs and other community health workers should be scaled up, particularly in low-literacy areas. Health education messages should emphasise early symptoms and the availability of free screening services. The Tobacco Free Educational Institute initiative under the National Tobacco Control Program should be strengthened to improve awareness of tobacco use in school-going children. Community-based interventions should be included in the program to also target non-school-going children in India. Opportunistic screening for oral cancer should be incorporated into routine healthcare visits, especially at primary health centers and during outreach camps. Training programs for frontline health workers and clinicians should be strengthened to ensure they are equipped to educate patients and conduct oral examinations. Additionally, consistent enforcement of existing tobacco control laws, including restrictions on surrogate advertising and point-of-sale promotion, must be reinforced. Finally, programmatic efforts should include regular monitoring and evaluation to assess awareness levels and screening coverage, thereby guiding improvements in intervention strategies.

### Strengths and limitations

This study has several strengths. It provides primary data from a vulnerable population directly affected by oral cancer, allowing for an in-depth understanding of their awareness and behaviour. The inclusion of both sociodemographic and behavioural factors enables a comprehensive analysis of potential determinants of awareness. Furthermore, the use of a pre-tested interview schedule and a structured data collection platform added consistency and reliability to the findings. However, the study also has limitations. It was conducted in a single tertiary care hospital in Delhi, limiting the generalisability of the results to other regions. Convenience sampling may have introduced selection bias and the exclusion of severely ill patients could have skewed the data away from those with the most advanced disease. As a cross-sectional study relying on self-reported data, the findings may also be affected by recall and social desirability bias. Despite these limitations, the study offers valuable insights that can inform targeted public health interventions aimed at reducing the burden of oral cancer in India.

## Conclusion

This study reveals significant gaps in awareness regarding tobacco-related oral cancer risks and preventive measures among patients with oral cancer. Despite high tobacco use, there is limited knowledge of early detection, government-led prevention programs and self-examination. Future studies should focus on studying the barriers to the implementation of the national programs and policies.

## Conflicts of interest

The authors have no competing interests to declare.

## Funding

The authors did not receive any funding for this work.

## Author contributions

PS – Conceptualisation, Data collection, Data analysis, Data interpretation, Manuscript – Original draft writing.

MMS – Conceptualisation, Data interpretation, Manuscript – Review and Editing, Supervision. ALB – Conceptualisation, Data interpretation, Manuscript – Review and Editing.

AS – Conceptualisation, Data interpretation, Manuscript – Review and Editing.

RM – Conceptualisation, Data interpretation, Manuscript – Review and Editing.
